# Glaucoma after ocular chemical burns: Incidence, risk factors, and outcome

**DOI:** 10.1038/s41598-020-61822-5

**Published:** 2020-03-16

**Authors:** Se Hyun Choi, Mee Kum Kim, Joo Youn Oh

**Affiliations:** 1 Department of Ophthalmology, Hallym University Sacred Heart Hospital, Anyang-si, Gyeonggi-do Republic of Korea; 2 Laboratory of Ocular Regenerative Medicine and Immunology, Seoul National University Hospital Biomedical Research Institute, Seoul, Korea; 3 Department of Ophthalmology, Seoul National University College of Medicine, Seoul, Korea

**Keywords:** Optic nerve diseases, Trauma

## Abstract

Effects of chemical injuries on the cornea and limbus have been widely studied; however, little is known about glaucoma after ocular chemical injuries. We herein investigated the incidence, risk factors, and outcome of glaucoma in patients with ocular chemical burns. Medical records were reviewed of patients who visited our clinic for chemical injuries to the ocular surface. Patients were divided into glaucoma and non-glaucoma groups based on high intraocular pressure (IOP) readings. Clinical characteristics, treatment method, and therapeutic and visual outcomes were compared between the two groups. Of 29 patients (40 eyes), 9 patients (15 eyes, 37.5%) were diagnosed with glaucoma at 2.64 ± 2.92 months after injury. Factors associated with glaucoma included male gender (p = 0.0114), bilateral ocular involvement (p = 0.0478), severe ocular surface involvement (Dua grades IV-VI, p = 0.0180), poor initial visual acuity (p = 0.0136), high initial IOP (p < 0.0001), pupil involvement at initial examination (p = 0.0051), and the need for amniotic membrane transplantation in the acute stage (p = 0.0079). At final follow-up, IOP was uncontrolled in 3 eyes (20.0%), and visual acuity was worse in the glaucoma group than in the non-glaucoma group (logMAR 2.94 ± 1.86 vs 0.34 ± 0.69, p < 0.0001). These findings suggest that careful evaluation and intensive treatment for glaucoma are essential in patients with severe ocular burns.

## Introduction

Chemical injuries to the eye induce a wide range of damage to ocular tissues, and severe alkali burns constitute a leading cause of intractable blindness^1,2^. Since the ocular surface is directly exposed to chemical injuries, most studies thus far have concentrated on investigating the effects of chemical injuries on the cornea and limbus, and the resulting corneal stromal scarring and limbal epithelial stem cell deficiency have been considered a major cause of vision loss in patients with ocular chemical burns^3–7^.

By contrast, posterior segment complications following chemical injuries to the ocular surface have been rarely investigated partly because of poor intraocular structure visualization in severely-burned eyes with corneal opacification. Recently, as the long-term restoration of corneal transparency can be achieved in eyes with severe chemical burns due to surgical advances in limbal cell transplantation and artificial cornea implantation, the unexpected observations of optic disc pallor and retinal degeneration have been reported^6,8–11^. These findings suggest that glaucoma and retinal damage are important sequelae of ocular chemical injuries. Few studies, however, have examined the incidence and clinical course of glaucoma in patients with ocular chemical burns^10,12–14^.

Herein, we investigated the incidence, onset, risk factors, and therapeutic and visual outcomes of glaucoma in patients with ocular chemical burns.

## Materials and methods

### Study design and subjects

This is a retrospective chart review study in patients who visited Seoul National University Hospital for ocular chemical burns between 2003 and 2017. The study was approved by the Institutional Review Board of Seoul National University Hospital (IRB No. H-1811-147-989) and performed in accordance with relevant guidelines and regulations. The need for informed consent was waived because the study involved only a retrospective chart review of anonymous patient data.

Patient inclusion criteria were as following: (1) patients who visited Seoul National University Hospital within 3 months of ocular chemical injuries and underwent thorough ophthalmic examination by ophthalmologists, (2) patients who were diagnosed with ocular burns and followed up for longer than one month after injuries.

### Data collection

The following data were obtained by the review of electronic medical charts and digital anterior segment photographs: demographic characteristics (age, sex, and race), onset and laterality of injuries, type of causative agents, best-corrected visual acuity (BCVA), intraocular pressure (IOP), severity of ocular burns as determined by both Roper-Hall and Dua classification systems^4,5^, medical and surgical managements of ocular surface burns, medical and surgical management of glaucoma, and duration of follow-up.

Digital anterior segment photographs serially taken in each patient during follow-up period were reviewed by a corneal specialist (S.H.C). From anterior segment photographs and medical records, ocular manifestations in acute and chronic stages were evaluated for the presence and extent of conjunctival and corneal epithelial defects, corneal stromal opacity, limbal ischemia, and involvement of iris, pupil, and lens. The pupil involvement was defined when the pupillary constriction in response to light was disturbed or a fixed, dilated pupil manifested. Based on these ocular manifestations, the severity of ocular burns was determined following Roper-Hall and Dua classification systems^4,5^. Grade I and II by Roper Hall classification and grades I to III by Dua classification were designated as low-grade burns, while grade III and IV by Roper Hall classification and grades IV to VI by Dua classification were categorized as high-grade burns.

The visual acuity recorded with Snellen charts was converted to logarithm of the Minimum Angle of Resolution (logMAR) equivalent using a conversion table for analysis^15^.

### Glaucoma diagnosis and data analysis

The diagnosis of glaucoma was made when a patient had IOP > 21 mmHg on more than two occasions after injury and required the anti-glaucoma treatment for IOP normalization during ocular chemical burn management. The IOP was measured primarily by Goldmann applanation tonometry, and if not possible, rebound tonometry (Icare PRO; Finland Oy, Helsinkin, Finland) was used.

Patients were divided into the following two groups: (1) patients who were diagnosed with glaucoma after injury (glaucoma group) and (2) those who did not show increased IOPs during the follow-up post-injury (non-glaucoma group). Demographical, ocular, and clinical data were compared between the two groups to identify factors associated with glaucoma development and to evaluate the therapeutic and visual outcomes of glaucoma.

### Statistical analysis

Statistical analysis was performed using GraphPad Software (GraphPad Prism, La Jolla, CA) and SPSS Software version 23.0 (SPSS, Inc., Chicago, IL). A nonparametric Mann-Whitney *U* test was used to compare quantitative variables between two groups, and Fisher’s exact test to compare qualitative variables. The independent risk factors affecting glaucoma development were determined by logistic regression and Cox regression analyses. Data were presented as the mean ± SD, and the difference was considered statistically significant at *p* < 0.05.

### Ethics approval

The Institutional Review Board of Seoul National University Hospital approved this study (IRB No. H-1811-147-989).

## Results

### Incidence and onset of glaucoma

A total of 65 patients visited Seoul National University Hospital for chemical injuries to the ocular surface between 2003 and 2017. Among them, 29 patients (40 eyes) met the inclusion criteria and were enrolled in the study.

Of 29 patients (40 eyes), 9 patients (15 eyes, 37.5%) had increased IOPs in the injured eye and required IOP-lowering treatments, and thereby were diagnosed of having glaucoma (Table 1). For comparative analysis, the 9 patients (15 eyes) developing glaucoma were included in the glaucoma group and the other 20 patients (25 eyes) in the non-glaucoma group. The mean follow-up durations were 41.5 ± 47.7 months (69.9 ± 62.9 months in the glaucoma group and 28.7 ± 33.8 months in the non-glaucoma group; *p* = 0.0981). Glaucoma was diagnosed in the glaucoma group at the mean 2.64 ± 2.92 months after onset of injury. At initial examination, the mean IOPs were 31.8 ± 10.9 mmHg in the glaucoma group and 14.7 ± 3.8 mmHg in the non-glaucoma group (*p* < 0.0001). The IOPs were high (>21 mmHg) from the initial examination in most of eyes (11 of 15 eyes, 73.3%) in the glaucoma group, whereas most of eyes (24 of 25 eyes, 96.0%) in the non-glaucoma group had normal IOPs at the initial examination.

**Table 1:** Demographics, clinical characteristics, and burn management.

	Total	Glaucoma group	Non-glaucoma group	*P* value
**Demographics and clinical characteristics**				
No. of patients (eyes)	29 (40)	9 (15)	20 (25)	
Age (years, range)	43.0 ± 14.8	41.3 ± 18.0	43.8 ± 13.6	0.8502
	(20–65)	(20–65)	(22–62)	
Sex (male:female)	19:10	9:0	10:10	0.0114
Laterality (unilateral:bilateral)	18:11	3:6	15:5	0.0478
Insulting agent (acid:alkali:unknown)	11:16:2	4:4:1	7:12:1	0.6754
Follow-up period (months, range)	41.5 ± 47.7	69.9 ± 62.9	28.7 ± 33.8	0.0981
	(1–185)	(1–185)	(1–129)	
**Ocular findings at initial presentation**				
Ocular burn severity				
Dua grade				
Low (I–III)	24	5	19	0.0180
High (IV–VI)	16	10	6	
Roper Hall grade				
Low (I–II)	17	4	13	0.1874
High (III–IV)	23	11	12	
Initial visual acuity (logMAR)	1.13 ± 1.07	1.63 ± 1.23	0.82 ± 0.85	0.0136
Initial IOP (mmHg)	20.9 ± 10.9	31.8 ± 10.9	14.7 ± 3.8	<0.0001
Initial IOP (normal:increased)	27:12	3:11	24:1	<0.0001
Corneal epithelial defect (No:Yes)	9:30	3:11	6:19	1.0000
Pupil involvement (No:Yes)	32:7	8:6	24:1	0.0051
**Acute management of ocular burn**				
IV steroid	2	2	0	0.0887
Topical corticosteroids	13	4	9	1.0000
Systemic steroid (IV and/or oral)	15	6	9	0.4270
Oral doxycycline	26	8	18	1.0000
Oral vitamin C	26	7	19	0.2200
Therapeutic contact lens	11:28	5:9	6:19	0.4780
Autologous serum eye drops	16:23	4:10	12:13	0.3171
AMT (No:Yes)	20:20	3:12	17:8	0.0079
**Chronic management of ocular burn**				
Penetrating keratoplasty (No:Yes)	29:11	7:8	22:3	0.0090
Limbal allogeneic graft (No:Yes)	31:9	8:7	23:2	0.0077
Buccal mucosa autograft (No:Yes)	35:5	12:3	23:2	0.3446
Eyelid surgery (No:Yes)	37:3	12:3	25:0	0.0461

logMAR: logarithm of the Minimum Angle of Resolution, IOP: intraocular pressure, IV: intravenous, AMT: amniotic membrane transplantation.

### Factors associated with glaucoma development

Demographical and ocular characteristics at initial presentation are summarized and compared between glaucoma and non-glaucoma groups in Table 1.

All patients were Koreans by ethnicity. The mean age at the time of injury was 43.0 ± 14.8 years (range, 20 to 65 years) and was not different between the glaucoma and non-glaucoma groups. Of 29 patients with ocular chemical burns, 19 patients were male and 10 were female. Remarkably, all 9 patients (100%) in the glaucoma group were male, whereas half of patients (10 out of 20 patients, 50%) were male in the non-glaucoma group (*p* = 0.0114). Overall, 11 of 29 patients (37.9%) were inflicted by chemical injuries in both eyes, and bilateral ocular involvement was more frequently found in the group with glaucoma (6 out of 9 patients, 66.7%), compared to the group without glaucoma (5 out of 20, 25.0%; *p* = 0.0478). Sixteen patients (55.2%) had alkali burns and 11 patients (37.9%) had acid burns, but there was no significant difference in the type of an insulting agent (alkali vs acid) between groups with and without glaucoma.

High grades of chemical burns as determined by Dua classification system (grades IV to VI) were significantly associated with glaucoma development. Ten out of 15 eyes (66.7%) in the glaucoma group had high-grade burns, as opposed to the finding that 6 out of 25 (24.0%) eyes in the non-glaucoma group had high grade burns (*p* = 0.0180). Similar observation was made with the visual acuity at initial examination. The initial visual acuity was 1.63 ± 1.23 logMAR values in the glaucoma group and 0.82 ± 0.85 logMAR values in the non-glaucoma group, and this difference was statistically significant (*p* = 0.0136).

Another notable finding was that the pupil involvement at initial presentation was more prevalent in eyes developing glaucoma than in those not developing glaucoma. The pupil involvement was observed in 6 out of 14 eyes (42.9%) in the glaucoma group. On the contrary, only one of 25 eyes (4.0%) in the non-glaucoma group had the pupil involvement at initial examination (*p* = 0.0051).

The acute management method for ocular chemical burns including topical or systemic corticosteroids was similar between glaucoma and non-glaucoma groups except for amniotic membrane transplantation (AMT) (Table 1). AMT was more commonly performed within 14 days of injury in eyes in the glaucoma group (12 of 15 eyes, 80.0%) than in those in the non-glaucoma group (8 of 25 eyes, 32.0%; *p* = 0.0079), an indication that the ocular surface burn was more severe in eyes developing glaucoma.

The surgical management for ocular surface burns in the chronic stage such as penetrating keratoplasty, allogeneic limbal transplantation, or eyelid reconstruction was more commonly carried out in the glaucoma group, another indication that the ocular surface was more severely affected by injury in the glaucoma group.

### Association of initial high IOP and pupil involvement with glaucoma

Logistic regression analysis was employed for identification of factors independently associated with glaucoma development after ocular chemical injury. As a consequence, an increased IOP (>21 mmHg) and pupil involvement at initial examination were found to be significantly associated with the development of glaucoma (*p* = 0.001 and 0.017, respectively) (Table 2). The odds ratios were 151.2 in eyes with high IOPs (>21 mmHg) at initial examination (vs eyes with IOPs ≤ 21 mmHg) and 43.4 in eyes with the pupil involvement (vs those without pupil involvement). Other factors such as sex, laterality of an injury, severity of ocular burn, or early AMT did not significantly affect the development of glaucoma (Table 2).

**Table 2:** Logistic regression analysis of risk factors for glaucoma development.

	Odds ratio	Confidence interval (95%)	*P* value
**Ocular findings at initial presentation**			
Increased IOP	151.247	8.508–2688.612	0.001
Pupil involvement	43.413	1.971–956.331	0.017
Ocular burn severity			0.444
Initial Visual Acuity			0.555
Corneal epithelial defect			0.872
**Demographics and clinical characteristics**			
Sex			0.312
Age			0.993
Laterality (unilateral or bilateral)			0.088
Insulting agent (acid or alkali)			0.299
**Acute management of ocular burn**			
Systemic steroid (IV and/or oral)			0.394
Oral doxycycline			0.615
Oral vitamin C			0.507
Therapeutic contact lens			0.983
Autologous serum eye drops			0.759

**Figure 1:**
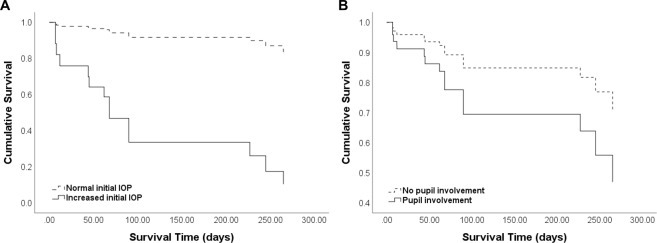
Survival curves from Cox regression analysis to determine the time from ocular chemical injury to glaucoma diagnosis. (**A**) During 6 months of follow-up, glaucoma developed in approximately 10% of patients who had normal intraocular pressures (IOP) at initial examination after injury, whereas glaucoma occurred in over 70% of patients whose IOP elevated to >21 mmHg at initial evaluation (p < 0.0001, B = 2.603). (**B**) Glaucoma developed in 85.7% of eyes (6 of 7) showing pupil involvement at initial examination and in 25.0% of eyes (8 of 32) without the initial pupil involvement. The time to glaucoma diagnosis was not different between eyes with and without pupil involvement (p = 0.189).

Furthermore, Cox regression analysis was applied to compare the time from injury to glaucoma diagnosis between eyes with and without high initial IOPs or between eyes with and without pupil involvement. The time-dependent Cox regression analysis revealed that an increased IOP at initial examination was a significant factor associated with glaucoma development (Fig. 1).

### Glaucoma management and outcome

The glaucoma management methods are depicted in Table 3. All 15 eyes (9 patients) in the glaucoma group were treated with topical anti-glaucoma medications. Oral acetazolamide was additionally used for IOP control in 7 patients (77.8%) and intravenous mannitol in 5 patients (55.6%). Four eyes (26.7%) received Ahmed glaucoma valve implantation at the mean 21.0 ± 20.9 months post-injury. Implant exposure occurred in 2 eyes (50.0%), one of which was treated by scleral patch graft and the other had a removal of the implant.

**Table 3:** Glaucoma management.

	No. (%)	Duration (days)
**Systemic medication**		
Intravenous mannitol	5 (55. 6)	
Oral acetazolamide	7 (77.8)	536 ± 1134
**Topical medication**		
β-blockers	11 (73.3)	940 ± 1359
Prostaglandin analogues	11 (73.3)	237 ± 475
α2-adrenergic agonists	14 (93.3)	885 ± 1180
Carbonic anhydrase inhibitors	10 (66.7)	755 ± 972
**Surgery**		
Ahmed valve implantation	4 (26.7)	21.0 ± 20.9 months (7, 10, 15, 52 months post-injury)

The final IOPs at the last follow-up were 20.8 ± 11.2 mmHg (range, 10 to 53 mmHg) in the glaucoma group and significantly higher than in the non-glaucoma group (13.7 ± 3.2 mmHg; *p* = 0.0086). Despite intensive medical and surgical managements, 3 eyes in the glaucoma group (20.0%) had high IOPs (>21 mmHg) at the last follow-up.

### Visual outcome

**Figure 2:**
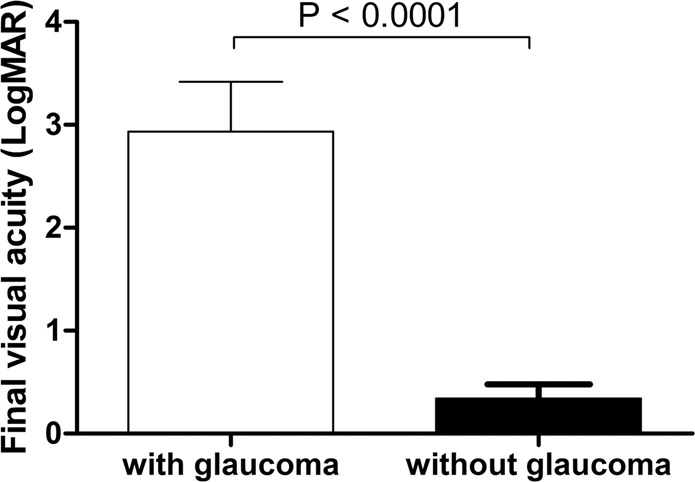
The final visual outcome in patients with ocular chemical burns according to the presence of glaucoma. The logarithm of the Minimum Angle of Resolution (logMAR) visual acuities were significantly poorer in eyes developing glaucoma than in those without glaucoma (p < 0.0001).

Overall, 20 of 40 eyes (50.0%) with ocular chemical injury achieved a Snellen BCVA of 20/40 or better at the final follow-up. Remarkably, the final visual acuity was significantly worse in patients in the glaucoma group compared to those in the non-glaucoma group. In the glaucoma group, 11 of 15 eyes (73.3%) had BCVA < 20/200 at the final visit, whereas only 3 of 25 eyes (12.0%) in the non-glaucoma group had a visual acuity < 20/200. The logMAR visual acuities were 2.94 ± 1.86 in eyes of the glaucoma group and 0.34 ± 0.69 in eyes of the non-glaucoma group (*p* < 0.0001) (Fig. 2).

Multiple linear regression analysis revealed that the presence of glaucoma (p < 0.0001), poor initial visual acuity (p = 0.009), need for limbal allogeneic transplantation (p = 0.002) were significant factors associated with poor final visual acuity in eyes with ocular chemical burns (R^2^ = 0.719).

Eventually, two eyes in the glaucoma group underwent evisceration at 22 days and 38 months after injury, respectively, whereas none were eviscerated in the non-glaucoma group.

## Discussion

Our data demonstrate that glaucoma developed in 37.5% of eyes with ocular chemical burns at the mean 2.64 months after injury. Male gender, bilateral ocular involvement, severe ocular surface involvement as determined by Dua grades IV to VI, poor visual acuity, high IOP and pupil involvement at the initial presentation, and the need for amniotic membrane transplantation in the acute stage were clinical factors observed more frequently in eyes developing glaucoma compared to those not developing glaucoma. Further regression analysis revealed that the risk of developing glaucoma was 151.2 times higher in eyes with initial IOPs > 21 mmHg (vs eyes with IOPs ≤ 21 mmHg) and 43.4 times higher in eyes with pupil involvement (vs eyes without pupil involvement) at initial examination.

A similar study by Lin *et al*.^12^ previously reported that ocular burn patients having severe ocular surface involvement as determined by higher Roper-Hall grades were more likely to require the long-term glaucoma treatment due to elevated IOP, as compared to patients without severe ocular surface involvement (84% vs 22%). In agreement with this finding, our results suggest that glaucoma was more frequent in eyes with high Roper-Hall grades than in those with low Roper-Hall grades although the difference did not reach statistical significance (47.8% vs 23.5%, *p* = 0.1874). In our patients, Dua grading system was better at predicting glaucoma development. Glaucoma was significantly more common in eyes with high Dua grades than in those with low Dua grades (62.5% vs 20.8%, *p* = 0.0180). This finding is consistent with a previous finding that the Dua classification system represents a better prognostic value in severe eye burns than the Roper-Hall system^16^. Our study is furthermore distinct from the study by Lin *et al*. in that we performed the stepwise comparison of a wide range of clinical characteristics between ocular burn patients with and without glaucoma for risk factor identification and outcome analysis, while Lin *et al*. described IOPs, percentage of eyes requiring glaucoma treatment, and visual acuities in mild (Roper-Hall grades I/II) vs severe ocular burns (Roper-Hall grades III/IV).

In our study, the final visual acuity was significantly poorer in eyes with glaucoma, compared to those without glaucoma. Also, an alternative analysis showed that glaucoma was one of prognostic factors determining the long-term visual outcome in patients with ocular chemical burns. These results collectively point to the critical importance of glaucoma prevention and treatment in the management of patients with ocular chemical burns. Despite the IOP control in 80% of glaucoma eyes by intensive management, the final BCVA was <20/200 in 73.3% of eyes with glaucoma, which presents the possibility of irreversible damage to the optic nerve and retinal ganglion cell layer after ocular surface burn. In this regard, several animal studies investigated the mechanism by which anterior burn could inflict harm on the posterior segment of an eye^17,18^. These studies revealed that the posterior segment damage was not mediated by direct detrimental effect of chemical agent or altered pH which was effectively buffered by the anterior segment. Rather, upregulation of pro-inflammatory mediators including tumor necrosis factor (TNF)-α caused retinal ganglion cell apoptosis and immune cell activation. The findings therefore suggest that prompt and aggressive anti-inflammatory treatment including corticosteroids and anti-TNF-α biologic agents could be a crucial adjunct to the current standard therapeutic modalities for ocular chemical burns and glaucoma^19,20^.

Our study has several limitations. First, glaucoma was defined only based on IOP measurement, and the detailed examination of optic nerve, retinal ganglion cell layer, and visual field was not possible because of corneal opacification. Second, the IOP values measured on opaque and irregular corneas might be unreliable. Nevertheless, the effects of high IOP on the visual outcome and association with other ocular findings were evident in our study. Further studies in a larger number of patients would strengthen the conclusions made in the present study.

When it comes to prognostic factors of ocular chemical burns, most studies have previously identified the severity of ocular surface involvement including corneal haze, limbal and conjunctival ischemia as major determinants of the visual outcome^4,5^. Our results additionally found that an elevated IOP and pupil involvement at initial examination were significantly associated with glaucoma development which is a critical limiting factor in the visual outcome in ocular burn patients. A careful ophthalmic evaluation for glaucoma and its risk factors during both initial examination and follow-up would allow for early detection and treatment of glaucoma and lead to vision preservation and rehabilitation in patients with severe ocular chemical burns

## Data Availability

The datasets used and/or analyzed during the current study are available from the corresponding author on reasonable request.
